# A new *Diancta* species of the family Diplommatinidae (Cyclophoroidea) from Vanua Levu Island, Fiji

**DOI:** 10.3897/zookeys.1073.73241

**Published:** 2021-11-29

**Authors:** Estée Bochud, David Haberthür, Ruslan Hlushchuk, Eike Neubert

**Affiliations:** 1 Natural History Museum of the Burgergemeinde Bern, Bernastrasse 15, CH-3005 Bern, Switzerland; 2 Institute of Ecology and Evolution, University of Bern, Baltzerstrasse 6, CH-3012 Bern, Switzerland; 3 Institute of Anatomy, University of Bern, Baltzerstrasse 2, CH-3012 Bern, Switzerland

**Keywords:** *Diancta*, Diplommatinidae, Fiji, new species, Oceania, terrestrial malacology

## Abstract

A new species of *Diancta* of the staircase snail family Diplommatinidae is described from Mt. Savusavu, Vanua Levu Island, Fiji. Due to its left coiling shell and a constriction before the last whorl, it is placed in the genus *Diancta*. Micro-CT imaging reveals two apertural teeth and an inner lamella that is situated at the zone of constriction. The shell abruptly changes coiling direction by 45 degrees before the last whorl. Up to now, this coiling modus had not yet been documented for any species of Diplommatinidae from the Fiji Islands.

## Introduction

Micromolluscs are defined being smaller than 5 mm and can be found in all parts of the world. They belong to different gastropod groups, are diverse in their habitat needs, appearance and, due to their small size, certainly underexplored. Still, many new species are found in all parts of the world. Usually, little is known about their ecology, distribution patterns and morphological variability. Because of their limited dispersal capabilities and microhabitat needs, microsnails demonstrate a high endemism rate. Terrestrial island snails especially show a high endemism rate of about 75% (Proios et al. 2021). Although the Diplommatinidae are distributed worldwide, there is one group among the terrestrial island micromolluscs that is particularly well represented in the Indo-Pacific region from Southeast Asia to the south-west Pacific and Australia (Stanisic et al. 2010). It is also one of the most locally and regionally diverse land snail families (Webster et al. 2012), showing high endemism. For example, from the Papuan and Wallacean region, 127 species are known (Greķe 2021), on Borneo nearly 170 diplommatinid taxa are found (Liew and Schilthuizen 2016), and on the Fiji islands 42 species had been documented so far (Neubert and Bouchet 2015).

Diplommatinids are known for having a zone of constriction close to the aperture (Egorov and Greķe 2003), internal teeth and lamellar structures (Thiele 1929), while some genera are characterized by a change in the coiling direction of the shell axis (Webster et al. 2012). The shell of the new species presented here is remarkable because it changes its coiling direction upwards to the apex and again back to its original coiling axis. This coiling mode was not yet known for any other species of Diplommatinidae from Fiji Islands.

This study is based on a dry sample collected by Otto Degener in 1941 on the island Vanua Levu of Fiji, previously housed in the MCZ collection (Cambridge, Massachusetts, USA). It aims to provide further information on the land snail richness of the Fiji Islands, particularly that of Vanua Levu, by describing this new diplommatinid species and providing the first diplommatinid record from western Vanua Levu. Dating from the Late Eocene, Vanua Levu is the second largest island within the Fiji Archipelago, which consists of more than 332 volcanic islands (Neall and Trewick 2008). As is known for other Fijian islands, some areas are covered by isolated limestone blocks presenting ideal ecological niches for Diplommatinidae. Despite its large area of 5807 square kilometres, only two localities of Vanua Levu are known for diplommatids, Waivunia village and Netewa Peninsula, from which nine species have been identified so far (Neubert and Bouchet 2015; Barker and Narosamalua 2017).

So far, the Fiji Islands are home to the diplommatinid genera *Diancta* E. von Martens, 1864, *Moussonia* O. Semper, 1865 and *Palaina* O. Semper, 1865 (Neubert and Bouchet 2015). We tentatively assign the new species to the genus *Diancta* based on the zone of constriction as described in Martens (1864) and in Kobelt’s (1902) emendation, “somewhat irregularly coiled”. As is the case with many Pacific islands, the Fiji Archipelago remains malacologically underexplored (Greke 2017). Phylogenetic data are underrepresented in the available data, and none of the Fijian Diplommatinidae has so far been molecularly assessed. Subsequently, it is not clear whether the unusual shape of the shell is simply a species-specific trait or whether it belongs to another genus.

Internal structures, such as the lamellae or plicae, were examined using X-ray microtomographic (micro-CT) imaging. Unfortunately, the shell broke during removal from the sample holder. Some dry remains of the animal itself could be found inside the shell. This mummified tissue could potentially be used for DNA extraction and sequencing.

## Methods

The description of this new species is based on a single dry shell from the type locality. There has been no living individual of this species collected or documented to date. Before scanning, the shell was manually cleaned of dried mud and moss with a fine brush and distilled water.

All different perspectives of the shell were captured using a Leica MC190 HD digital camera connected to a Leica M205 C stereo microscope (Leica Microsystems GmbH, Wetzlar, Germany). The multifocal images were processed using the Leica proprietary software LAS X EDOF version 3.6.0.20104 (Leica Microsystems).

Micro-CT was conducted at the Anatomical Institute in Bern, Switzerland. The sample was mounted in a small custom-made cylindrical sample holder (3D-printed: https://git.io/Jc4De) and imaged on a Bruker SkyScan 1272 high-resolution microtomography machine (Control software version 1.4, Bruker microCT, Kontich, Belgium). The X-ray source was set to a tube voltage of 50.0 kV and a tube current of 200.0 µA, and the sample was imaged with an unfiltered x-ray spectrum. A set of 322 projection images of 1632 × 1092 pixels were taken at every 0.6° over a 180° recorded sample rotation. Every single projection was exposed for 339 ms. Three projections were averaged to reduce image noise. This resulted in a scan time of approximately 16 minutes. The projection images were then reconstructed into a 3D stack of images with NRecon (Version 2.0.0.5, Bruker microCT, Kontich Belgium). The whole process resulted in a dataset with an isometric voxel size of 7.5 µm. The 3D images and videos were visualized using the CTvox software Version 3.3.1 (Bruker microCT) and the Image J software version 1.53c 2020.

The raw data from the micro-CT scan as well as the reconstructions are-in the spirit of reproducible research-available online (Haberthür et al. 2021): https://doi.org/10.17605/OSF.IO/CSGKQ.

Measurements were made using the LAS X software measuring tool and are given in mm. Abbreviations used are: SH = shell height, SW = shell width, AH = aperture height, AW = aperture width, W = number of whorls after Kerney et al. (1983).

### Museum abbreviations

**NMBE** Natural History Museum of the Burgergemeinde Bern, Bern, Switzerland

**MCZ**Museum of Comparative Zoology, Harvard University, Cambridge, Massachusetts, USA.

## Systematic part

### Diplommatinidae L. Pfeiffer, 1856

#### Genus Diancta

Taxon classificationAnimaliaArchitaenioglossaDiplommatinidae

E. v. Martens, 1864

AF264970-AD92-58AD-8E77-A25A88903CA0

Diancta E. v. Martens, 1864: Type species: Diplommatinaconstricta Martens, 1864 [Moluccas, Indonesia].

##### Diagnosis.

This species is placed in the genus *Diancta* because of the sinistral shell, constriction of the shell and closed umbilicus (Neubert and Bouchet 2015).

#### Diancta phoenix

Taxon classificationAnimaliaArchitaenioglossaDiplommatinidae

Bochud sp. nov.

F6BA401D-9CF2-5214-A227-0BEFC77053B8

http://zoobank.org/12242324-0720-46A5-A9D6-0253E6140F10

**Figure 1A, B:**
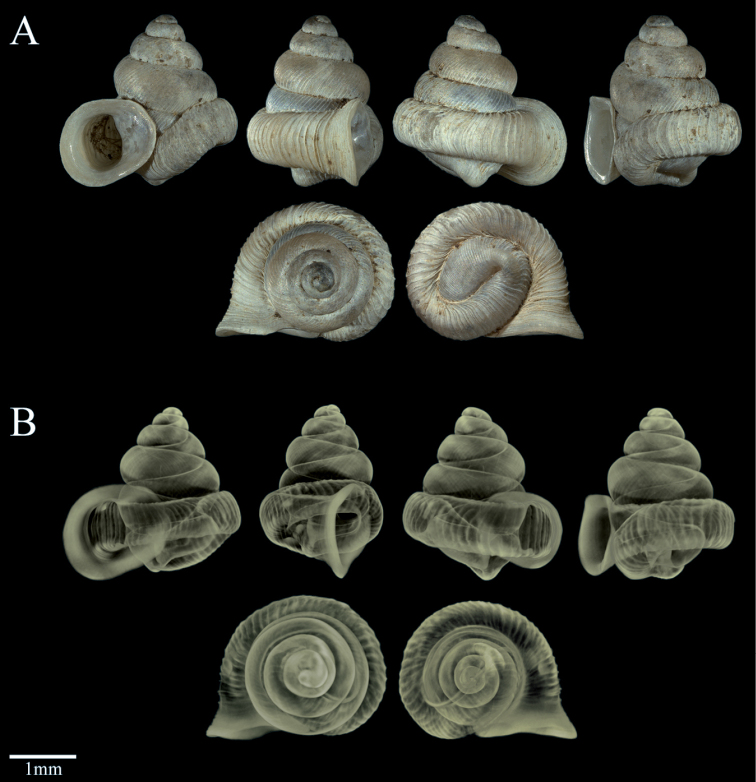
*Dianctaphoenix* sp. nov. Holotype, MCZ 394198, SH = 2.59 mm **A** Different external views of the shell **B** Micro-CT views.

**Figure 2A, B:**
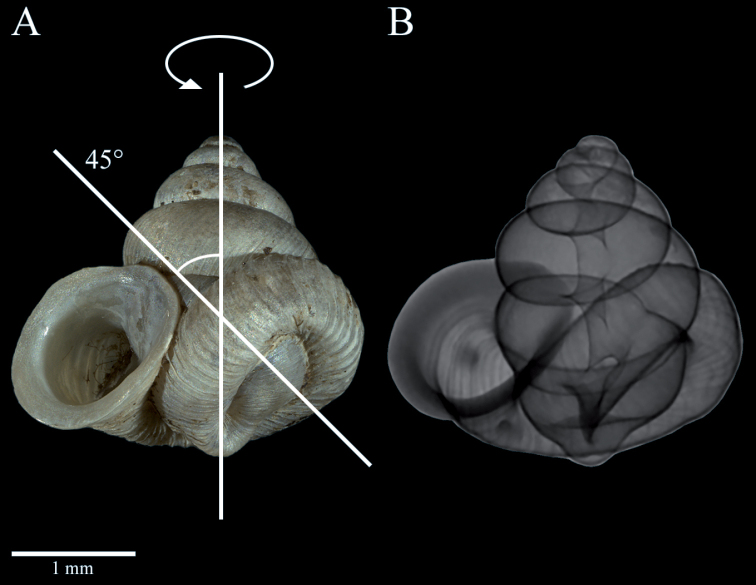
*Dianctaphoenix* sp. nov., change in coiling axis by 45 degrees **A** Tilted view of the left coiled shell **B** Micro-CT picture, visualisation of the columella.

**Figure 3A–D:**
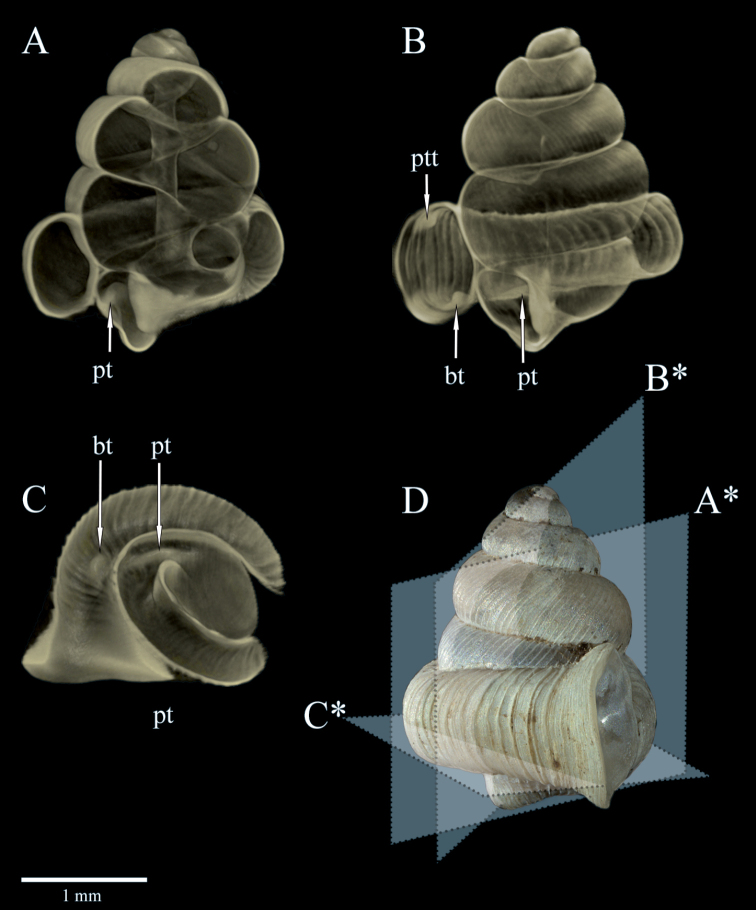
*Dianctaphoenix* sp. nov., Micro-CT scans **A** Zone of constriction with palatalis = pt **B** Apertural teeth; palatal tooth (ptt) and basal tooth (bt) and palatalis **C** Additional view of the basal teeth and parietalis **D** Sectional planes through the shell to the corresponding letters A*–C*.

**Figure 4:**
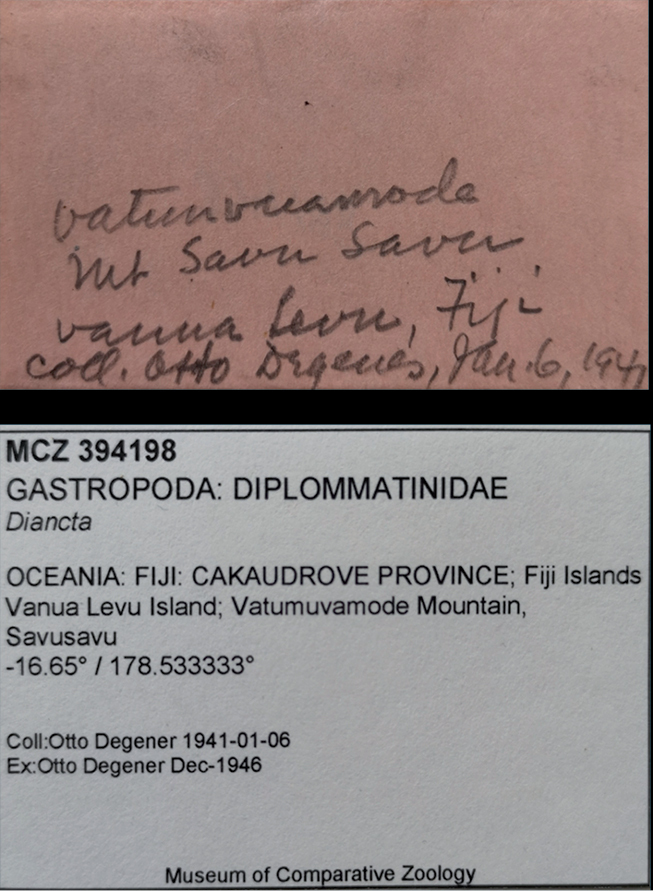
original label from 1941 by Otto Degener and the interpreted label by MCZ, Harvard.

Figs 1 -4 , Suppl material 1 -3

##### Type locality.

Fiji, Cakaudrove Province, Vanua Levu Island, Vatumuvamode Mountain, Savusavu, -16.65°N, 178.53°E 63 m a.s.l. (original label text).

##### Type material.

***Holotype*.**MCZ 394198 ex coll. Otto Degener., leg. Otto Degener, 6.1.1941. 1 shell, SH = 2.59, SW = 2.85, AH = 1.34, AW = 1.57, W = 6.25. The protoconch and peristome are the only remaining parts of the broken shell.

##### Etymology.

The new species is named after the immortal saga bird that arises from its ashes. The species epithet is derived from the bird’s name: Phoenix. It is a noun in apposition. Despite the broken holotype, this species is being kept “alive” by pictures, 3D prints and Micro-CT scans.

##### Description.

shell sinistral, tiny (SH = 2.59 mm); pyramidal shaped; consisting of 6.25 whorls separated by a shallow suture; protoconch dull, smooth, 2.5 whorls; surface of teleoconch shell with radially aligned, regularly and finely formed axial ribs; ribs slightly curved; last whorl bears sharper ribs, ribbing pattern less regular, with a larger spacing between ribs; whorls rapidly increasing in size, shell constricted after four whorls; constriction site prominent, forming a bulge situated one whorl behind the aperture at the umbilicus; change of coiling axis after zone of constriction, turning the shell 45 degrees upward towards the apex; aperture large, about half shell height, slightly oval shaped and attached to the shell; two visible teeth located in the aperture; one small upper palatal tooth, and opposite a somewhat elongated basal tooth; peristome simple and continuous; inside shell, above ventral bulge, with an elongated palatal lamella opposite the very narrow constriction; columellar plate reduced; umbilicus closed.

##### Distribution.

This species is known so far only from the type locality.

##### Remarks.

According to the original label (Fig. 4), the shell was found in a place interpreted as Vatumuvamode on Mount Savu Savu. Close to the city of Savusavu in the South of Vanua Levu, there is a hill named Suva Suva. On topographic Fijian maps dated 1985, this hill is called mount Nasuvasuva (352 m a.s.l.). We were unable to allocate the mountain Savu Savu or a place called Vatumuvamode. It is difficult to say whether Degener’s Savu Savu is a misspelling of Nasuvasuva, or whether he meant another place. The exact meaning of Vatumuvamode is also unclear. In the northwestern part of the island, a place called Savu Sau exists. A path leads from there to the Vuadomo waterfalls, reminiscent of Vatumuvamode. Since the shell was found in 1941 during World War II, and at a period when Fiji was a British colony, it is very likely that names of the places changed since then, or it belongs to an old village or defence site that is not shown on maps. However, the label and its interpretation seem contradictory and unresolvable. Additional sampling of more localities is needed to locate the exact type locality of the new species.

The shell was already quite eroded. The boundary between the protoconch and teleoconch is not clearly visible, while several ribs are partially removed or degraded. There is also no recognizable colouring on the shell. Because other shell specimens and living animals of this species are lacking, it raises the question whether the specimen studied could be an aberrant shell of a species that has already been described. In any case, it is not possible to perform a comparative study on the morphology of shells, operculum, radula, and/or genitalia.

Other genera of Diplommatinidae with a directional change of the coiling axis include *Moussonia* O. Semper, 1865, *Opisthostoma* W. T. Blanford & H. F. Blanford, 1860, *Plectostoma* H. Adams, 1865, and *Whittenia* T.-S. Liew & Clements, 2020. *Moussoniamonstrificabilis* Greķe, 2017 changes coiling direction from dextral to sinistral, which is not the case in the newly described species. The aperture in *Opisthostoma* points towards the apex or the dorsal side of the shell due to an alteration in the coiling axis (Nurinsiyah and Hausdorf 2017). This is not seen in *Dianctaphoenix* sp. nov. In addition, the doubled peristome, mentioned in the original description of *Opisthostoma* by Blanford (1860), is missing. Usually, *Plectostoma* has a detached last whorl (Kobelt 1902; Egorov 2013; Liew et al. 2014b) and an “extraordinary prolongation backwards of the free portion” (Adams 1865). This is not the case for *D.phoenix* sp. nov. Liew and Gopalasamy (2020) described the new genus *Whittenia*, which conchologically resembles *Opisthostoma* but differs by the outer whorl being raised above the level of the apex, and distinguishing it also from our specimen. None of these character state combinations applies to the new species. In contrast, the penultimate whorl of the shell is constricted, as originally described by von Martens (1864) for the genus *Diancta*. Due to the upward bend in coiling, the last whorl wraps once again around the constricted whorl and gives a pyramidal appearance to the shell. In *Opisthostoma* and *Plectostoma*, the coiling axis changes, but, more importantly, the aperture ends detached from the shell or points in an upwards or other direction to that of the shell axis. For this reason, we assign the new species to *Diancta*.

##### Differential diagnosis.

**Figure 5A–D:**
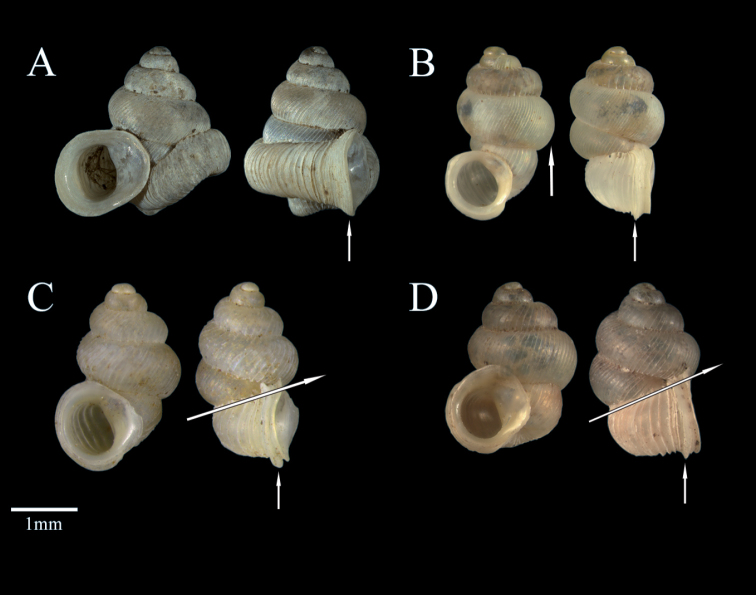
Comparison of *D.phoenix* sp. nov. with other Fijian species **A***D.phoenix* sp. nov., with simple peristome, SH = 2.59 mm **B***D.rotunda* Neubert and Bouchet, 2015, with bulbous penultimate whorl, SH = 2.65 mm **C***D.macrostoma* (Mousson, 1870), SH = 2.84 mm and **D***D.martensi* (H. Adams, 1866), SH = 2.62 mm, with strong ascending last whorl and double peristome.

Applying the key of Neubert and Bouchet (2015) to the new species, identification attempts lead to the species *D.rotunda* Neubert & Bouchet, 2015, due to the sinistral shell and reduced columellar plate. This species has a small palatal fold deep in the aperture and a shell height of 2.6 mm in the same size range as *D.phoenix* sp. nov., but it clearly differs by its quite bulbous penultimate whorl. Other species that are similar in size are *D.macrostoma* (Mousson, 1870) and *D.martensi* (H. Adams, 1866). With their strongly ascending last whorl, these two species are reminiscent of an incipient change in the coiling axis, as is the case for the newly described species. However, the missing shell features in the new species are the alteration of the coiling axis and the presence of frontally visible apertural teeth. The peristome of *Dianctaphoenix* sp. nov. is simple and not doubled as in the other described species (Fig. 5).

Several species of Fijian *Diancta*, like *D.macrostoma* and *D.martensi*, share a strong ascending last whorl and a similar ribbing pattern. *Dianctaphoenix* sp. nov. is distinguished from all Fijian species by the clear coiling axis twist of 45 degrees, the presence of a simple peristome, the umbilical bulge, its simple columella and the two teeth present in the aperture. To evaluate the variability of these traits, more specimens must be sampled. Changes in the coiling axis are documented for different snail groups and seem to have independently evolved several times (Páll-Gergely and Neubauer 2020).

## Discussion

Diplommatinidae are mainly still assessed using shell characters. The original descriptions of the three genera from Fiji are quite short and have been emended via additional shell characters by subsequent authors such as Kobelt (1902) and Egorov (2013) The classification into genera, subgenera and species has already been regarded as difficult when focusing only on shell characters (Rundell 2008; Webster et al. 2012; Neubert and Bouchet 2015). For example, Köhler and Kessner (2020) found a high variation in shell ribbing in a single population of *Diplommatinafluminis* B. Rensch, 1931. Many species are only known from a single shell or a limited number of specimens, which hampers any serious conclusions about the variability of shell morphology. The risk that the specimen described herein is an aberrant form must be considered, due to the lack of comparative material from the type locality. However, the probability of finding a new species is quite high, considering that Diplommatinidae are very small in size, are local endemics and have only been documented in three localities from three regions of Vanua Levu. Clements et al. (2008) mentioned a mutant form, but also that the intraspecific variation among shell dimensions seems to be low. Therefore, we conclude that it is more probable to have a new species rather than an abnormal form, considering the clear differences in shell morphology compared to previously described species in Fiji.

For further sampling of fresh material, it is necessary to explore the northwestern part of the island in the Savu Sau region, as well as the Savu Savu mountain in the central-southern region, to find out exactly where the new species is found. The assignment to the genus *Diancta* is tentative. Here, the inclusion of the type species of the genus *Diancta*, *Diplommatinaconstricta* Martens, 1864, from the Moluccas in Indonesia, would be mandatory to confirm this generic assignment. Micro-CT is a highly useful and seldomly used method for revealing important diagnostic characters such as the inner dentition and the lamellae, especially in micromolluscs, which are difficult to handle. This method was malacologically pioneered and successfully used for assessing inner shell characters and variability in the genera *Plecostoma* and *Opisthostoma* (Liew et al. 2014a; Liew and Schilthuizen 2016), and far surpasses the need to break rare and valuable shells to expose internal structures (Budha et al. 2017).

## Supplementary Material

XML Treatment for Diancta

XML Treatment for Diancta phoenix

## Supplementary materials

Supplementary material 1 Movie 1 Data type media Explanation note 360-degrees rotatable 3D shell model of *D.phoenix*. https://binary.pensoft.net/file/615270This dataset is made available under the Open Database License (http://opendatacommons.org/licenses/odbl/1.0/). The Open Database License (ODbL) is a license agreement intended to allow users to freely share, modify, and use this Dataset while maintaining this same freedom for others, provided that the original source and author(s) are credited.David Haberthür

Supplementary material 2 Movie 2 Data type media Explanation note Sagittal slicing through the 3D shell model of *D.phoenix* showing internal teeth and zone of constriction. https://binary.pensoft.net/file/615271This dataset is made available under the Open Database License (http://opendatacommons.org/licenses/odbl/1.0/). The Open Database License (ODbL) is a license agreement intended to allow users to freely share, modify, and use this Dataset while maintaining this same freedom for others, provided that the original source and author(s) are credited.Estée Bochud

Supplementary material 3 Movie 3 Data type media Explanation note Transparent 3D model of *D.phoenix* rotating upside down showing internal teeth, columella, ventral bulge and zone of constriction. https://binary.pensoft.net/file/615272This dataset is made available under the Open Database License (http://opendatacommons.org/licenses/odbl/1.0/). The Open Database License (ODbL) is a license agreement intended to allow users to freely share, modify, and use this Dataset while maintaining this same freedom for others, provided that the original source and author(s) are credited.Estée Bochud
